# Supplementary material for: Vaccine confidence and hesitancy among mothers of children under six years of age in Salvador, Brazil: The role of sociodemographic factors and health service experience
Source: PLoS One. 2026 May 15;21(5):e0344742. doi: 10.1371/journal.pone.0344742 (PMC13178900; doi:10.1371/journal.pone.0344742)
Supplement: S1 File — (DOCX) [file pone.0344742.s001.docx]

**
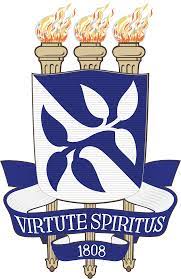
FEDERAL UNIVERSITY OF BAHIA**

**NURSING SCHOOL**

**POSTGRADUATE PROGRAM IN NURSING AND HEALTH**

**RESEARCH TITLE: DIMENSIONS OF IMPACT OF TERRITORY ON THE HEALTH AND NUTRITION CONDITIONS OF CHILDREN IN EARLY CHILDHOOD**

| Date: | Time: | UBS: | Interviewer: |
| --- | --- | --- | --- |

**BLOCK 1 – GENERAL IDENTIFICATION OF MOTHER AND CHILD**

| Mother's name |  |
| --- | --- |
| Mother's age |  |
| Child's name |  |
| Child's date of birth |  |
| Complete address |  |
| Contact phone number/WhatsApp |  |
| Mother's race/color | 1. Brown ( ) 2. Black ( ) 3. White ( ) 4. Yellow ( ) 5. Indigenous ( ) |
| Race/Color of the child | 1. Brown ( ) 2. Black ( ) 3. White ( ) 4. Yellow ( ) 5. Indigenous ( ) |
| What grade did you study until? |  |
| Do you live with a partner? | 1. Yes, he is the child's father ( ) 2. Yes, but he is not the child's father ( ) 3. No ( ) |
| Do you have any religion? | 1. Evangelical ( ) 2. Candomblé ( ) 3. Catholic ( ) 4. Other ( ) 5. None ( ) |
| Do you have any profession or technical training? | 1. Yes ( ) Which one?__________________________________ 2. No ( ) |
| Are you currently working? | 1. Formal employment (CLT/Concurso) 2. Informal employment/odd jobs 3. I'm not working ( ) |
| Type of housing | 1. House ( ) 2. Apartment ( ) 3. Farm/country house ( ) |
| Housing conditions | 1. Running water ( ) 2. Sewage/garbage collection ( ) 3. Electricity ( )  4. Raising animals for consumption ( ) 5. Home gardening ( ) |
| Number of people living in the household (adults/children) | Adults:_________________ Children/teenagers (up to 18 years old):_________ |
| Who do you consider to be the head of the family? | 1.Mother ( ) 2.Father ( ) 3.Other ( )__________________________ |
| Do you participate in any Income Transfer Program? (Auxílio Brasil, Others) | 1. Yes ( ) Which one?__________________________________ 2. No ( ) |
| Do you or your child have health insurance? | 1. Yes ( ) 2. No ( ) |
| Family income (DO NOT consider government assistance) |  |

**BLOCK 2 - MOTHER'S HEALTH AND NUTRITION CONDITIONS**

| Do you have any chronic illnesses? | 1. Hypertension ( ) 2. Diabetes ( ) 3. History of maternal depression ( )  4. Smoker ( ) 5. Others ( )___________________________________________ | | |
| --- | --- | --- | --- |
| You had prenatal care. | 1. Yes ( ) Month prenatal care started:_______________ Number of consultations:______________  2.No ( ) | | |
| Type of delivery | 1.Vaginal ( ) 2.Cesarean ( ) | Prematurity: 1. Yes ( ) 2. No ( ) |  |
| Did you have maternity leave? | 1. Yes ( ) 2. No ( ) 3. Not applicable ( ) | | |
| Were you diagnosed with syphilis during pregnancy? | 1. Yes, and I did the treatment ( ) 2. Yes, but I did not do the treatment ( ) 3. No ( ) 4. I don't know ( ) | |  |
| Was the child diagnosed with congenital syphilis at birth? | 1. Yes ( ) 2. No ( ) 3. Don't know ( ) | |  |
| How old were you when you became pregnant for the first time? |  | |  |
| **FOOD CONSUMPTION MARKERS** | | |  |
| Do you usually eat your meals while watching TV, using the computer and/or cell phone? 1. Yes ( ) 2. No ( ) | | |  |
| What meals do you usually eat throughout the day? | ( ) Breakfast ( ) Morning snack ( ) Lunch ( ) Afternoon snack ( ) Dinner ( ) Supper | |  |
| Yesterday, you consumed: | ( ) Bean  ( ) Fresh fruit (do not consider fruit juice)  ( ) Vegetables and/or legumes (do not consider potatoes, cassava, cassava, yam and taro)  ( ) Hamburger and/or sausages (ham, mortadella, salami, sausage, hot dogs)  ( ) Sweetened beverages (soda, carton juice, powdered juice, carton coconut water, guarana/blackcurrant syrups, fruit juice with added sugar)  ( ) Instant noodles, packaged snacks or salty crackers  ( ) Stuffed biscuits, sweets or treats (candies, lollipops, gum, caramel, jelly) | |  |
| Do you dislike or have an allergy/intolerance to any food? | 1. Yes ( ) Which one?______________________________ 2. No ( ) | |  |
| **PERCEPTION OF BODY IMAGE AND NUTRITIONAL STATUS** | | |  |
| Which of these figures is most like you? SHOW STUNKARD SILHOUETTE SCALE | Put the number of the picture here _____________ | |  |
| And which of these figures would you like to look like? SHOW STUNKARD SILHOUETTE SCALE | Put the number of the picture here _____________ | |  |
| Stunkard Silhouette Scale (Stunkard, Sorensen, & Schulsinger, 1983)  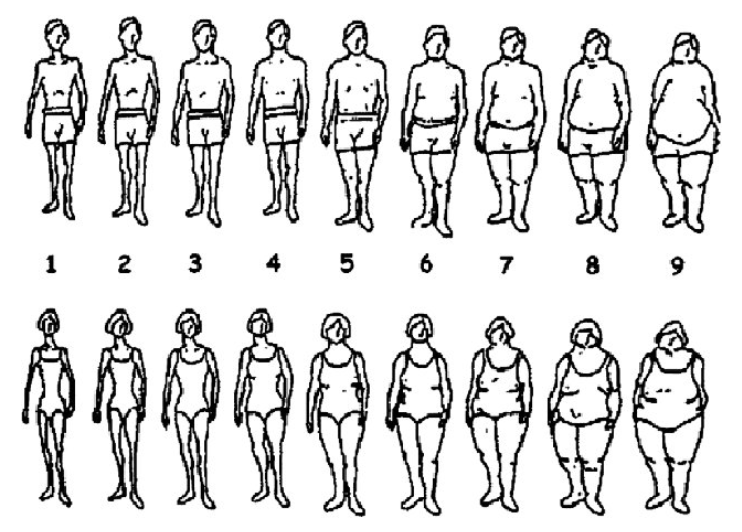 | | |  |
| WEIGHT: _________ Kg Height:_______________________ m | | |  |
| **MATERNAL MENTAL HEALTH – SYMPTOMS RELATED TO THE LAST 30 DAYS** | | |  |

| Do you have frequent headaches? | 1. Yes ( ) 2. No ( ) 3. Don't know/ didn't answer ( ) |
| --- | --- |
| Do you have a lack of appetite? | 1. Yes ( ) 2. No ( ) 3. Don't know/ didn't answer ( ) |
| Do you sleep badly? | 1. Yes ( ) 2. No ( ) 3. Don't know/ didn't answer ( ) |
| Do you get scared easily? | 1. Yes ( ) 2. No ( ) 3. Don't know/ didn't answer ( ) |
| Are your hands shaking? | 1. Yes ( ) 2. No ( ) 3. Don't know/ didn't answer ( ) |
| Do you feel nervous, tense or worried? | 1. Yes ( ) 2. No ( ) 3. Don't know/ didn't answer ( ) |
| Is your digestion not good or do you suffer from digestive disorders? | 1. Yes ( ) 2. No ( ) 3. Don't know/ didn't answer ( ) |
| Can't you think clearly? | 1. Yes ( ) 2. No ( ) 3. Don't know/ didn't answer ( ) |
| Are you feeling unhappy? | 1. Yes ( ) 2. No ( ) 3. Don't know/ didn't answer ( ) |
| Do you cry more than usual? | 1. Yes ( ) 2. No ( ) 3. Don't know/ didn't answer ( ) |
| Do you find it difficult to enjoy (enjoy) your daily activities? | 1. Yes ( ) 2. No ( ) 3. Don't know/ didn't answer ( ) |
| Do you find it difficult to make decisions? | 1. Yes ( ) 2. No ( ) 3. Don't know/ didn't answer ( ) |
| Is your daily work a pain? A torment? Do you have difficulty doing your job? | 1. Yes ( ) 2. No ( ) 3. Don't know/ didn't answer ( ) |
| Do you feel like you are not capable of playing a useful role in life? | 1. Yes ( ) 2. No ( ) 3. Don't know/ didn't answer ( ) |
| Have you lost interest in things? | 1. Yes ( ) 2. No ( ) 3. Don't know/ didn't answer ( ) |
| Do you think you are a worthless person? | 1. Yes ( ) 2. No ( ) 3. Don't know/ didn't answer ( ) |
| Has the thought of ending your life ever crossed your mind? | 1. Yes ( ) 2. No ( ) 3. Don't know/ didn't answer ( ) |
| Do you feel tired all the time? | 1. Yes ( ) 2. No ( ) 3. Don't know/ didn't answer ( ) |
| Do you have unpleasant sensations in your stomach? | 1. Yes ( ) 2. No ( ) 3. Don't know/ didn't answer ( ) |
| Do you get tired easily? | 1. Yes ( ) 2. No ( ) 3. Don't know/ didn't answer ( ) |

**BLOCK 3 – CHILDREN’S HEALTH AND NUTRITION CONDITIONS**

| Child's sex: ( ) Male ( ) Female | | Birth order of child/number of children: ______/________ | | |  |
| --- | --- | --- | --- | --- | --- |
| Birth weight: | Apgar 1'_____ 5'_____ | | Did you breastfeed in the first hour of life? 1. Yes ( ) 2. No ( ) | |  |
| Did you have a heel prick test when you were born? 1. Yes ( ) 2. No ( ) 3. Don't know/don't remember | | | | |  |
| Did you have your tongue tested when you were born? 1. Yes ( ) 2. No ( ) 3. Don't know/don't remember | | | | |  |
| Did you have a heart test when you were born? 1. Yes ( ) 2. No ( ) 3. Don't know/don't remember | | | | |  |
| Did you have an eye test when you were born? 1. Yes ( ) 2. No ( ) 3. Don't know/don't remember | | | | |  |
| Did you have your hearing tested when you were born? 1. Yes ( ) 2. No ( ) 3. Don't know/don't remember | | | | |  |
| Did any of the tests above show any changes? 1. Yes ( ) Which one?____________________ 2. No ( ) 3. Don't know/don't remember ( ) | | | | |  |
| Did you receive a home visit from a health professional in the first week of life? 1. Yes ( ) 2. No ( ) 3. Don't know/don't remember ( ) | | | | |  |
| Does the child have any health problems? 1. Yes ( ) 2. No ( ) Which ones? | | | | |  |
| Does the child have sickle cell anemia? 1. Yes ( ) 2. No ( ) 3. Don't know | | | | |  |
| Has the child been hospitalized in the last 15 days? 1. Yes ( ) Reason_____________________ 2. No ( ) | | | | |  |
| the child have COVID-19? 1. Yes ( ) 2. No ( ) 3. Don't know | | | | |  |
| **CHILD HEALTH MONITORING** | | | | |  |
| Does the child receive routine check-ups at the neighborhood health unit? 1. Yes ( ) 2. No ( ) | | | | |  |
| Do you receive home visits from health professionals? 1. Yes ( ) 2. No ( ) | | | | |  |
| How far is it from your home to the Health Unit? 1. Up to 500m (two blocks) 2. From 500 to 1000m (2 to 4 blocks) 3. More than 1000m | | | | |  |
| Do health professionals usually ask about the child's diet? 1. Yes ( ) 2. No ( ) | | | | |  |
| Do health professionals often ask about a child's development? 1. Yes ( ) 2. No ( ) | | | | |  |
| Do you usually participate in health education groups about child care? 1. Yes ( ) 2. No ( ) | | | | |  |
| Does the child receive or have received Ferrous Sulfate prescribed by a health professional? 1. Yes ( ) 2. No ( ) | | | | |  |
| Does the child receive or has he/she received a megadose of Vitamin A every 6 months? 1. Yes ( ) 2. No ( ) | | | | |  |
| How would you rate the quality of the health services your health unit offers to you and your child?  1. Excellent ( ) 2. Good ( ) 3. Reasonable ( ) 4. Bad ( ) 5. Indifferent ( ) 6. Does not use the service ( ) | | | | |  |
| How do you consider your relationship with the health professionals at your health unit?  1. Excellent ( ) 2. Good ( ) 3. Reasonable ( ) 4. Bad ( ) 5. Indifferent ( ) 6. Does not use the service ( ) | | | | |  |
| Do you use the internet to take care of your child's health?  ( ) I look for information on Google about taking care of the child  ( ) I participate in communities on social media to take care of the child  ( ) I carry out online/telemedicine consultations  ( ) I receive messages via WhatsApp from healthcare professionals  ( ) I don't use/have internet | | | | |  |
| **VACCINE HESITATION** | | | | |  |
| **In the options below, answer whether you:**  **1 2 3 4 5**  **disagree disagree nor agree agree strongly agree**  **strongly and neither disagree**  ( ) Vaccines are important for my child's health  ( ) Vaccines work  ( ) Vaccinating my child is important for the health of other children in my neighborhood.  ( ) All childhood vaccines that are provided by the government are beneficial  ( ) New vaccines present more risks than old ones  ( ) I trust the information I received from professionals about vaccines.  ( ) Vaccinating is a good way to protect my child from diseases.  ( ) I generally follow the vaccination guidelines recommended by the health professionals who care for my child.  ( ) I worry about serious reactions to vaccines  ( ) My child does not need vaccines for diseases that are no longer common today. | | | | |  |
| Some people do not believe in vaccination, do you agree with these groups? 1. Yes ( ) 2. No ( ) 3. Partially ( ) | | | | |  |
| When a new vaccine is provided at the health unit, you:  ( ) Take your child to get vaccinated soon ( ) Prefer to wait to see how other people will react to the vaccine ( ) Don't vaccinate | | | | |  |
| Have you ever stopped vaccinating your child because of some information you received? (There may be more than one option)  ( ) No ( ) Health professional ( ) Internet ( ) Television/radio/newspapers ( ) Relatives or friends | | | | |  |
| Have you ever purposely delayed a vaccine or decided not to get vaccinated? ? 1. Yes ( ) 2. No ( ) 3. Don't remember | | | | |  |
| If yes, what is the reason? (There may be more than 1 option)  ( ) Fear of reactions ( ) Health Unit closed  ( ) Vaccination is not necessary ( ) The child has already had any adverse events following vaccination  ( ) Religious reasons  ( ) Difficulty accessing health services for vaccination (too far away) ( ) There was a lack of vaccines at the health unit  ( ) Very long waiting time ( ) Medical contraindication  ( ) Was not instructed to vaccinate ( ) Several injections at the same time  ( ) Lack of information regarding contraindications (child was sick).  ( ) Lack of time ( ) Didn't like a health service professional  ( ) Own choice/Did not want to vaccinate the child | | | | |  |
| **EVALUATION OF THE CHILD'S RECORD** | | | | |  |
| Does the child have a Child's Record from the Ministry of Health? 1. Yes ( ) 2. No ( ) | | | | |  |
| Have you read the Child's Notebook? 1. Completely ( ) 2. Partially ( ) 3. I haven't/never read it ( ) | | | | |  |
| Is there any weight/height record in the growth charts in the Child's Record?  1. Complete ( ) 2. Incomplete ( ) 3. There is no record ( ) 4. The booklet is not in hand ( ) | | | | |  |
| Is there any record of child development monitoring in the Child's Record?  1. Complete ( ) 2. Incomplete ( ) 3. There is no record ( ) 4. The booklet is not in hand ( ) | | | | |  |
| Regarding the vaccination schedule: 1. Complete ( ) 2. Incomplete ( ) 3. ( ) The booklet is not in hand | | | | |  |
| Is there any record of the administration of Megadose of Vitamin A in the Child's Record?  1. Complete ( ) 2. Incomplete ( ) 3. There is no record ( ) 4. ( ) The notebook is not in hand | | | | |  |
| Is there any record of the supply of Ferrous Sulfate in the Children's Booklet?  1. Complete ( ) 2. Incomplete ( ) 3. There is no record ( ) 4. ( ) The notebook is not in hand | | | | |  |
| **EATING PRACTICES AND NUTRITIONAL STATUS** | | | | |  |
| Is the child breastfeeding or was he/she breastfeeding? 1. Yes ( ) 2. No ( ) | | | | |  |
| Until what age did the child breastfeed? ______________________ ( ) Still breastfeeds ( ) Never breastfed | | | | |  |
| At what age did the child start receiving water/tea/bottle? ______________________________ ( ) I don't remember | | | | |  |
| At what age did the child start receiving food from the family (pot-cooked meals)? _________ ( ) I don't remember ( ) Not applicable | | | | |  |
| Does the child dislike or have an intolerance to any food? 1. Yes ( ) Which one?___________________________ 2. No ( ) | | | | |  |
| Does the child have the habit of eating meals while watching TV, using the computer and/or cell phone? 1. Yes ( ) 2. No ( ) | | | | | |
| YESTERDAY, what meals did the child eat throughout the day?  1.Breakfast ( ) 2.Morning snack ( ) 3.Lunch ( ) 4.Afternoon snack ( ) 5.Dinner ( ) 6.Supper ( ) | | | | | |
| YESTERDAY, the child consumed: 1. Beans ( ) 2. Fruits ( ) 3. Vegetables and/or legumes ( ) 4. Cereals/tubers ( ) 5. Meat/Eggs ( ) 6. Milk and dairy products ( ) 7. Soft drinks/sweetened beverages ( ) 8. hamburgers and/or cold cuts ( ) 9. instant noodles, packaged snacks or crackers ( ) 10. stuffed biscuits, sweets or treats ( ) | | | | | |
| Who usually prepares meals in your house? | | | | | |
| Which of the pictures is most like your child? ____________________ | | | | |  |
| What figure would you like the child to look like? _______________________ | | | | |  |
| 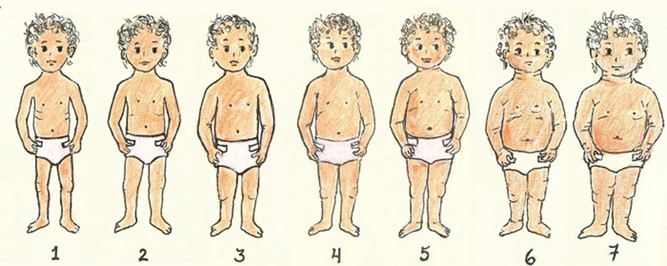Children's Body Image Silhouette Scale (HAGER et al, 2010) | | | | |  |
| Current weight: ______________ g Current height: ______________cm | | | | Hemoglobin level:______________g/dL |  |
| **CHILD DEVELOPMENT** | | | | |  |
| Does your child attend daycare/preschool? 1. Public ( ) 2. Private ( ) 3. No ( ) | | | | |  |
| Does the child have toys at home? 1. Yes ( ) 2. No ( ) | | | | |  |
| Do you have children's books at home? 1. Yes ( ) 2. No ( ) | | | | |  |
| Does your house/apartment have a yard/playground or any space for the child to play? 1. Yes ( ) 2. No ( ) | | | | |  |
| Have you done any of these activities with your child in the last three days? 1. Read/tell stories ( ) 2. Sing ( ) 3. Play ( )  4. Take for a walk ( ) 5. Name things/count/draw ( ) 6. None( ) | | | | |  |
| Do you often use punitive discipline to educate your child (yelling, grounding and/or hitting)?  1. Always ( ) 2. Often ( ) 3. Sometimes ( ) 4. Almost never ( ) 5. Never ( ) | | | | | |
| When your child cries, how do you usually react?  ( )Try to calm down immediately (pick up, talk)  ( ) Let him cry for a while and then try to calm him down.  ( ) Ignore it and let it cry until it stops on its own  ( ) She gets irritated and calls the child's attention. | | | | | |
| SWYC Infant Development Scale: scale number:______________ score:___________ | | | | | |
| **SCALE OF CONFLICT BETWEEN PARENT AND CHILDREN (the questions refer to some time in life)** | | | | | |
| Did you explain to the child why what he/she was doing was wrong? 1. Yes ( ) 2. No ( ) | | | | | |
| Did you punish him/her by telling him/her to stay in his/her room or somewhere else? 1. Yes ( ) 2. No ( ) | | | | | |
| Did you shake the child? 1. Yes ( ) 2. No ( ) | | | | | |
| Did you hit his/her butt with a belt, flip-flop, hairbrush, stick or other hard object? 1. Yes ( ) 2. No ( ) | | | | | |
| Did you give him/her something else to do instead of what he/she was doing wrong? 1. Yes ( ) 2. No ( ) | | | | | |
| Did you speak loudly, yell, or scream at the child? 1. Yes ( ) 2. No ( ) | | | | | |
| Did you hit him/her with a closed hand or kick him/her hard? 1. Yes ( ) 2. No ( ) | | | | | |
| Did you spank his/her butt? 1. Yes ( ) 2. No ( ) | | | | | |
| Did you grab him/her by the neck and shake him/her? 1. Yes ( ) 2. No ( ) | | | | | |
| Did you curse or swear, that is, curse him/her? 1. Yes ( ) 2. No ( ) | | | | | |
| Did you hit the child a lot, that is, did you hit him/her non-stop, as much as you could? 1. Yes ( ) 2. No ( ) | | | | | |
| Have you ever said that you were going to kick him/her out of the house or chase him/her out of the house? 1. Yes ( ) 2. No ( ) | | | | | |
| Did you burn the child or spill hot liquid on him or her on purpose? 1. Yes ( ) 2. No ( ) | | | | | |
| Did you threaten to slap him/her, but didn't? 1. Yes ( ) 2. No ( ) | | | | | |
| Did you hit any part of his body other than his buttocks with a belt, flip-flop, stick, or other hard object? 1. Yes ( ) 2. No ( ) | | | | | |
| Did you slap the child's hand, arm, or leg? 1. Yes ( ) 2. No ( ) | | | | | |
| Did you take away his/her perks or leave him/her homeless? 1. Yes ( ) 2. No ( ) | | | | | |
| Did you pinch the child? 1. Yes ( ) 2. No ( ) | | | | | |
| Did you threaten him/her with a knife or gun? 1. Yes ( ) 2. No ( ) | | | | | |
| Did you throw the child on the floor? 1. Yes ( ) 2. No ( ) | | | | | |
| Did you call him/her stupid, dumb, lazy, or something similar? 1. Yes ( ) 2. No ( ) | | | | | |
| Did you slap the child on the face, head or ears? 1. Yes ( ) 2. No ( ) | | | | | |

**BLOCK 4. HOUSEHOLD FOOD AND NUTRITIONAL SECURITY**

| In the past three months, have the residents of this household been concerned that food would run out before they could buy or receive more food? | 1. Yes ( ) 2. No ( )  3. I don't know/ I didn't answer ( ) |
| --- | --- |
| In the last three months, has food run out before the residents of this household had money to buy more food? | 1. Yes ( ) 2. No ( )  3. I don't know/ I didn't answer ( ) |
| In the last three months, have the residents of this household run out of money to eat a healthy and varied diet? | 1. Yes ( ) 2. No ( )  3. I don't know/ I didn't answer ( ) |
| In the last three months, the residents of this household have eaten only a few types of food they still had because they ran out of money? | 1. Yes ( ) 2. No ( )  3. I don't know/ I didn't answer ( ) |
| In the last three months, has any resident aged 18 or over missed a meal because there was no money to buy food? | 1. Yes ( ) 2. No ( )  3. I don't know/ I didn't answer ( ) |
| In the last three months, has any resident aged 18 or over ever eaten less than they thought they should because there was no money to buy food? | 1. Yes ( ) 2. No ( )  3. I don't know/ I didn't answer ( ) |
| In the last three months, has any resident aged 18 or over ever felt hungry but did not eat because there was no money to buy food? | 1. Yes ( ) 2. No ( )  3. I don't know/ I didn't answer ( ) |
| In the last three months, has any resident aged 18 or over ever eaten only one meal a day or gone a whole day without eating because there was no money to buy food? | 1. Yes ( ) 2. No ( )  3. I don't know/ I didn't answer ( ) |

**BLOCK 5. DIMENSIONS OF THE TERRITORY ( Early Childhood Friendly Neighborhood: safe, green and free, accessible, playful, inclusive)**

| In your neighborhood, are leisure areas located safely and easily accessible? 1. Yes ( ) 2. No ( ) |
| --- |
| Do you feel safe walking or cycling around your neighborhood? 1. Yes ( ) 2. No ( ) |
| Do you think your neighborhood has play spaces or active and attractive facades for children? 1. Yes ( ) 2. No ( ) |
| Are there adequate and well-located bus stops in your neighborhood? 1. Yes ( ) 2. No ( ) |
| Are there adequate parking areas in your neighborhood that do not interfere with pedestrians? 1. Yes ( ) 2. No ( ) |
| Do the sidewalks in your neighborhood have ramps for baby strollers? 1. Yes ( ) 2. No ( ) |
| Are there adequate street lighting in your neighborhood? 1. Yes ( ) 2. No ( ) |
| Are there bike paths in your neighborhood? 1. Yes ( ) 2. No ( ) |
| In your neighborhood, are streets usually closed for events/street markets? 1. Yes ( ) 2. No ( ) |
| In your neighborhood, do you have easy access to services such as shops, schools, daycare, health centers, community centers, etc.?  1. Yes ( ) 2. No ( ) |
| Are the streets in your neighborhood tree-lined? 1. Yes ( ) 2. No ( ) |
| Is your neighborhood very noisy? 1. Yes ( ) 2. No ( ) |
| Is it common to have floods in your neighborhood? 1. Yes ( ) 2. No ( ) |
| In your neighborhood, do you see a lot of trash on the street/open sewers? 1. Yes ( ) 2. No ( ) |
| In your neighborhood, do you feel safe because your neighbors, relatives, and known business owners form a trustworthy community network and are generally involved and attentive to the child? 1. Yes ( ) 2. No ( ) |
