# Supplementary material for: Vaccine confidence and hesitancy among mothers of children under six years of age in Salvador, Brazil: The role of sociodemographic factors and health service experience
Source: PLoS One. 2026 May 15;21(5):e0344742. doi: 10.1371/journal.pone.0344742 (PMC13178900; doi:10.1371/journal.pone.0344742)
Supplement: Attachment — Submitted filename: PONE-D-25-23001_revKC150625.pdf [file pone.0344742.s003.pdf]

Dear Authors,

Congratulations on your article. The theme is original.

However, I will need to make some suggestions to increase the chance of the article being published.

Abstract: OK

Background:
In Lines 62-64(Vaccine hesitancy, the delay in acceptance or refusal of vaccines despite availability, has become a significant public health challenge, contributing to declining immunization coverage worldwide), get the literature references about this.

Methods:

-Why did the authors decide to use 2010 IBGE, if there are data from 2022 IBGE and SINASC 2022 for children, and if the study is from 2023?
- Line 112- 113 - How did they calculate the 95% confidence
level, 5% margin of error, to add the 20% to account for potential losses?
- Why were children with neurological diseases excluded?
- If the mothers were interviewed, was it a questionnaire or forms? Have validation? Was it an adaptation from the other validated instrument of data collection? Is this instrument present in the supplementary annex of the article?
- The Inclusion and Exclusion Criteria section should be more details.
- L138-145: The parameters about the Vaccine Hesitancy Scale need to be put in a square.
- Ethical considerations should be the last section.
- Statistical analyses are not clear. Is the population a normal curve? Or is non-parametric analyses there are needs? Why didn't the authors decide on a regression model?
-See: How to Write Your Methods - PLOS. available in https://plos.org/resource/how-to-write-your-methods/?utm_medium=email&utm_source=internal&utm_campaign=modnewsletters&utm_content=modnewsletter.
- See: Akel KB, Masters NB, Shih SF, Lu Y, Wagner AL. Modification of a vaccine hesitancy scale for use in adult vaccinations in the United States and China. Hum Vaccin Immunother. 2021 Aug 3;17(8):2639-2646. doi: 10.1080/21645515.2021.1884476. Epub 2021 Mar 26. PMID: 33769209; PMCID: PMC8475604.
-See: Daniel Kotz, Jochen W.L. Cals, Effective writing and publishing scientific papers, part IV: methods, Journal of Clinical Epidemiology,
Volume 66, Issue 8,2013, Page 817, ISSN 0895-4356,
https://doi.org/10.1016/j.jclinepi.2013.01.003.(https://www.sciencedirect.com/science/article/pii/S089543561300019X)

Results
- The authors should improve the presentation of results.

- See: Kotz, D., & Cals, J. W. (2013). Effective writing and publishing scientific papers, part V: results. Journal of Clinical Epidemiology, 66(9), 945. https://doi.org/10.1016/j.jclinepi.2013.04.003
-See: Duquia RP, Bastos JL, Bonamigo RR, González-Chica DA, Martínez-Mesa J. Presenting data in tables and charts. An Bras Dermatol. 2014;89(2):280-285. doi:10.1590/abd1806-4841.20143388
-See: Daniel Kotz, nJochen W.L. Cals. Effective writing and publishing scientific papers, part VII: tables and figures. WRITING TIPS SERIES|VOLUME 66, ISSUE 11, P1197, NOVEMBER 01, 2013 Open AccessPublished:August 19, 2013DOI:https://doi.org/10.1016/j.jclinepi.2013.04.016

Discuss
- Rewrite according to alterations in Methods and results.
- See: Cals, J. W., & Kotz, D. (2013). Effective writing and publishing scientific papers, part VI: discussion. Journal of Clinical Epidemiology, 66(10), 1064. https://doi.org/10.1016/j.jclinepi.2013.04.017
