# Supplementary material for: Vaccine confidence and hesitancy among mothers of children under six years of age in Salvador, Brazil: The role of sociodemographic factors and health service experience
Source: PLoS One. 2026 May 15;21(5):e0344742. doi: 10.1371/journal.pone.0344742 (PMC13178900; doi:10.1371/journal.pone.0344742)
Supplement: Attachment — Submitted filename: Response to reviewers.docx [file pone.0344742.s004.docx]

**Response to Reviewers – Manuscript PONE-D-25-23001**

**Title: Confidence and Vaccine Hesitancy Among Mothers of Children Under Six Years Old in Salvador, Brazil: The Role of Sociodemographic Factors and Experience with Health Services**

Dear Academic Editor,
Dear Reviewers,

We sincerely thank you for your thoughtful comments and valuable suggestions, which have significantly contributed to the improvement of our manuscript. Below, we present our point-by-point responses, indicating the changes made or justifications provided for each observation.

Reviewer #1 – Dr. Karen Cordovil

1. Lines 62–64 – Reference on vaccine hesitancy:
   ✔️ We have included updated references supporting the statement regarding the impact of vaccine hesitancy on global immunization coverage.
2. Use of IBGE 2010 data:
   ✔️ We have justified the use of 2010 data within the manuscript.
3. Sample calculation (lines 112–113):
   ✔️ We have detailed the sample calculation process.
4. Exclusion of children with neurological conditions:
   ✔️ We clarified that this exclusion aimed to avoid bias in maternal perceptions of vaccination, given the specific clinical history of these children.
5. Data collection instrument:
   ✔️ We specified that a structured questionnaire was used, adapted from a previously validated instrument. The full version has been included as a supplementary appendix.
6. Inclusion/exclusion criteria:
   ✔️ This section was expanded to include detailed information on the criteria applied.
7. Lines 138–145 – Vaccine Hesitancy Scale:
   ✔️ The parameters were organized into a table to facilitate visualization.
8. Ethical considerations:
   ✔️ We repositioned this section to the end of the Methods, as suggested.
9. Statistical analyses:
   ✔️ We clarified that data distribution was assessed and, as it did not follow a normal curve, non-parametric tests were used.

Reviewer #2 – Dr. Márcio Cristiano de Melo

1. Discussion – Examples of interventions:
   ✔️ We included examples of outreach strategies, community health worker involvement, and digital initiatives, as suggested.
2. Appendix with adapted scale:
   ✔️ The final version of the 10-item scale, translated and applied, was included as an appendix.
3. Implications for public policy:
   ✔️ We added a final paragraph in the Discussion addressing practical implications for immunization policy development.

Reviewer #3 – Anonymous

1. Line 62 – Reference on vaccine hesitancy:
   ✔️ Reference added.
2. Line 88 – Larger study:
   ✔️ We named the main study and included the corresponding reference.
3. Line 156 – Mean and standard deviation:
   ✔️ We clarified that these were used only for continuous variables.
4. Line 165 – Origin of the question:
   ✔️ We indicated that the question is part of the applied scale. The complete questionnaire was included as an appendix.
5. Line 175 – Variables in initial analysis:
   ✔️ We listed all variables used and referenced the corresponding table.
6. Line 212 – Construction of factors:
   ✔️ We detailed the process of constructing the “lack of confidence” and “risk perception” factors based on exploratory factor analysis.
7. Lines 317 and 323 – Fake news and misinformation:
   ✔️ We rewrote these sections to align with the study data and included references supporting the discussion on misinformation.
8. Line 338 – User profile bias:
   ✔️ We included a reflection on the profile of health service users and its implications for sample representativeness.

We reiterate our gratitude for the opportunity to improve this work. We hope the revisions meet the expectations of the editorial team and reviewers.

Sincerely,
Claudia Palombo and co-authors
