# Supplementary material for: Vaccine confidence and hesitancy among mothers of children under six years of age in Salvador, Brazil: The role of sociodemographic factors and health service experience
Source: PLoS One. 2026 May 15;21(5):e0344742. doi: 10.1371/journal.pone.0344742 (PMC13178900; doi:10.1371/journal.pone.0344742)
Supplement: Attachment — Submitted filename: Response to reviewers2_.docx [file pone.0344742.s005.docx]

**PONE-D-25-23001R1**

Dear Dr. Yakum,

We sincerely thank you and the reviewers for the constructive comments on our manuscript entitled *“Confidence and hesitancy regarding vaccination among mothers of children under six years in Salvador, Brazil: the role of sociodemographic factors and experiences with health services.”* We greatly value the opportunity to revise and resubmit our work to *PLOS ONE*.

Below, we present our point-by-point responses:

1. Ethical statement
   The manuscript has been revised to explicitly state that informed consent was obtained from the parents or legal guardians of the participating children. We clarified that assent does not apply to children under six years of age, in accordance with standard ethical terminology in research.
2. Statistical analysis
   In response to Reviewer #2, we included explicit results of the R² values to better convey the explanatory power of the models.
3. Writing style
   Minor editorial adjustments were made to eliminate repetitions in the final paragraphs and to enhance the overall clarity of the text.

We believe that these revisions address all concerns raised and strengthen the manuscript. We are grateful for the reviewers’ recognition of its scientific merit and relevance to public health policy.

We look forward to the consideration of our revised submission.

Sincerely,
Dr. Palombo and co-authors
